# Neobractatin mediated ZBP1 induces PANoptosis of xenografted breast tumors

**DOI:** 10.3389/fphar.2026.1913482

**Published:** 2026-09-09

**Authors:** Ying Guo, Chao Li, Kidong Eom, Jing Dong, Lin Li

**Affiliations:** 1 College of Animal Science &Veterinary Medicine, Shenyang Agricultural University, Shenyang, Liaoning Province, China; 2 Key Laboratory of Livestock Infectious Diseases, Ministry of Education, Shenyang Agricultural University, Shenyang, Liaoning Province, China; 3 Department Veterinary Diagnostic Imaging, College of Veterinary Medicine, Konkuk University, Seoul, Republic of Korea

**Keywords:** breast cancer, food as medicine, natural compound, neobractatin, panoptosis

## Abstract

Breast cancer is the most common malignant tumor in women. Garcinia bracteata fruit is a plant considered to possess high nutritional and medicinal value and is edible in its raw state. Neobractatin (NBT) is a natural compound isolated from Garcinia bracteata. In this study, we select EMT6 cells to establish an EMT6 mouse breast cancer model, and to investigate the tumor suppression effect of NBT *in vitro* and *in vivo*. CCK-8, cell cloning, flow measurement of cell cycle and apoptosis, LDH, ROS, MMP, q-PCR, WB, HE staining and transcriptome sequencing were used in this study. We find that NBT effectively reduces the viability of EMT6 cells and causes cell cycle arrest. *In vivo* experiments have shown that the survival status of mice has generally improved, and the tumor volume has significantly decreased with NBT. NBT ameliorates breast cancer progression by ZBP1-mediated PANoptosis *in vivo* and *in vitro*. This study aims to provide a theoretical basis for the development of NBT as a novel anti-breast tumor drug.

## 1 Introduction

The incidence of breast cancer is increasing globally, and in 2020, data show that breast cancer has surpassed lung cancer as the most common cancer death in the world (Lei et al., 2021; Loibl et al., 2024). According to historical cancer data, breast cancer accounts for nearly one-fifth of all female cancer cases (Sung et al., 2021; Nagahashi and Miyoshi, 2024). In actual clinical practice, patients with early-stage breast cancer remain at high risk of recurrence after treatment (DeSantis et al., 2017; AlSendi et al., 2021; Pedersen et al., 2022). Despite rapid advances in breast oncology and the development of multiple precision therapies for breast cancer, its high degree of heterogeneity continues to make it one of the leading causes of cancer-related deaths among women (Xiong et al., 2025). Meanwhile, as the cancer progresses, distant organ metastasis is also one of the biggest challenges faced by breast cancer patients in clinical treatment. Relevant studies have shown that breast cancer is the second most common cause of brain metastasis onset in patients, with the cerebellum accounting for 33% of cases (De Luca et al., 2023). The lungs are also one of the target organs where breast cancer is prone to metastasis. In Western countries, distant organ metastasis of breast cancer has become the second leading cause of death for cancer patients. In the absence of effective response strategies, it is predicted that the global incidence of new breast cancers will peak by 2070 and breast cancer will become an essential public health problem worldwide (Soerjomataram and Bray, 2021). Medicinal foods derived from herbs offer high safety and minimal side effects. For example, mushroom intake, together with other phytotherapy substances, protects against cancer, specifically gastrointestinal (GI) and breast cancers. Furthermore, mycotherapy has a significant positive impact on the long-term survival rate, tumor response, host immune function, inflammation, and quality of life of cancer patients (Rossi et al., 2018). The search for food-derived drugs with excellent therapeutic efficacy and low toxicity is one of the hotspots in breast cancer research today.

A new form of inflammatory cell death, panoptosis, was proposed in 2019. When cells undergo necrotic apoptosis, pyroptosis and necroptosis superimposed on each other, a concept of total cell death called PANoptosis is formed (Shi et al., 2023). Current research indicates that PANoptosis can be regulated by key factors such as ZBP1 and RIPK3 modulate caspase-dependent and caspase-independent cascades (Wang W. Q. et al., 2025). The PANoptosis score is significantly associated with the tumor microenvironment, the infiltration levels of most immune cells (namely, NK cells, CD8^+^ T cells, CD4^+^ T cells, and DCs), and immune-related genes (Zhang et al., 2024). One study analyzed breast cancer profiles using the TCGA and GEO databases and found that high expression of PANoptosis was beneficial in reducing the incidence of breast cancer (Liu et al., 2024).

Garcinia bracteata fruit is a plant considered to possess high nutritional and medicinal value and is edible in its raw state (Tan et al., 2025; Zheng et al., 2022). It exhibits multiple pharmacological effects, including antibacterial, anti-inflammatory, antiviral, antioxidant, and antitumor properties (Tan et al., 2022). The caged xanthones Neobractatin (NBT) extracted from the 95% ethanol extract of Garcinia bracteata have excellent anti-tumor activity. NBT can lead to the blockage of G1/S and G2/M phases during proliferation and division of HeLa cells by affecting the expression of E2F1 and GADD45α, and thereby exerting a tumor-suppressive effect (Zheng et al., 2019). In addition, NBT can also inhibit the proliferation of HeLa and K562 cells by regulating the expression of RNA-binding protein CELF6 (Zheng et al., 2022). In the study of esophageal cancer, NBT, on the other hand, inhibits the proliferation of esophageal cancer cells through GSDME-mediated pyroptosis (Tan et al., 2022). In metastasis studies of tumor cells, NBT can inhibit the metastasis of breast and lung cancer cells by upregulating MBNL2 expression and mediating the pAKT/EMT signaling pathway (Zhang et al., 2019). However, the potential mechanism by which neobractatin inhibits breast cancer remains unclear.

In this study, we found that NBT induces PANoptosis in EMT6 cells. *In vivo* experiments, we found that NBT inhibits breast tumor growth by inducing PANoptosis. In addition, we used the natural chemotherapeutic agent paclitaxel as a positive control and conducted a preliminary assessment of its *in vivo* safety. This experiment will provide some theoretical basis for future treatment and drug screening of clinical breast cancer.

## 2 Materials and methods

### 2.1 Cell culture

EMT6 cells were purchased from ATCC and cultured in 1640 (Sigma, United States) containing 10% fetal bovine serum (FBS, Life Technologies, United States) and 1% Penicillin-Streptomycin Solution (Life Technologies, United States) for incubation, 5% CO2, 37 °C. The experiments were divided into DMSO group (1% DMSO), NBT-L group (2.4 μM NBT), NBT-M group (3.0 μM NBT), and NBT-H group (3.6 μM NBT).

### 2.2 Cell siRNA transfection

Cells were cultured in six-well plates, and transfection was performed when cells were in good condition. 1750 mL OPTI-MEM(Glpbio, Montclair,American) was added to the six-well plate, and then a mixture of si-Zbp1 and transfected liposomes was added to the transfection wells to make the final concentration of siRNA 100 nM. At 6 h after transfection, the culture medium was changed to DMEM containing 10% fetal bovine serum, and protein assay was performed at 48 h. Assess the knockdown efficiency using Western blotting.

### 2.3 CCK-8

The CCK8 kit was purchased from Elabscience (China). EMT6 cells were inoculated in 96-well plates at 5 × $10^{3}$ /well, and incubated at 37 °C in a 5% CO2, and dosing was carried out when the cells in the wells were adhered to the wall and grew to 70%–80%. The NBT were treated with the EMT6 cells for 24 h and 48 h. The cell proliferation was detected by adding 10 μL of CCK8 solution per well for 1.5 h. The OD value at 450 nm was measured by an enzyme marker.

### 2.4 Lactate dehydrogenase (LDH) release assay

The LDH kit was purchased from Beyotime (China). EMT6 cells were inoculated in 96-well plates at 5× $10^{3}$ /well. Incubated at 37 °C, 5% CO2, and then dosed when the cell adherence in the culture wells grew up to 70%–80%, and the NBT were treated for 24 h. The cell supernatant centrifugate at 1000r for 5 min. Pipette 120 μL of cell supernatant and add 60 μL of LDH assay working solution. Incubate for 30 min at room temperature and avoid light, and measure the absorbance value at 490 nm.

### 2.5 Cell morphology observation

EMT6 cells were evenly spread in 6-well plates at a number of 2.5 × $10^{6}$ cells/well, and when the cells in each well grew to 70%–80% adherent to the wall, the cells were treated with different concentrations of NBT for 24 h. Morphological changes of EMT6 cells were directly observed under a microscope, and the observations were photographed.

### 2.6 Clonogenic assay

EMT6 cells were homogeneously inoculated in 6-well plates at 5× $10^{2}$ /well for 24 h, and NBT were treated for 24 h. After removing the drugs in the wells and washing, cell culture medium was added to each well to continue cultivation for 10–14 days. The cell culture medium was replaced every 3 days. Each well was sequentially fixed with 2 mL of 4% paraformaldehyde for 30 min, and stained with 1 mL of crystal violet for 10 min. Images were obtained after natural drying.

### 2.7 Mitochondrial membrane potential detection

EMT6 cells were evenly spread in 6-well plates at the number of 2.5× $10^{6}$ /well, and treated with NBT after growth to 70%–80%. The medium of drug and positive reagent group was removed, and 1 mL of cell culture medium was added. 1 mL of JC-1 staining working solution was added to each well and incubated at 37 °C for 20 min. At the end of incubation, the cells were washed twice with JC-1 staining buffer (1×). After adding 2 mL cell culture solution was processed under fluorescence microscope for photographs, and the cells need to be digested before loading JC-1 probe for quantitative detection by flow cytometry.

### 2.8 Reactive oxygen species (ROS) detection

EMT6 cells were evenly spread in 6-well plates according to the number of 2.5 × $10^{6}$ /well, and then treated with NBT after growth to 70%–80%. Positive control wells and negative control wells were set. Wash the cells once with Reagent III working solution, then add 1 mL of Reagent I working solution. Incubate at 37 °C in the dark for 50 min. Wash three times with Reagent III working solution, then add Reagent III working solution. Observe under a fluorescence microscope and process photographs.

### 2.9 Apoptosis detection

EMT6 cells were spread in 6-well plates according to the number of 2.5× $10^{6}$ /well, and treated with NBT after growth to 70%–80% for 24 h. The cells were digested with 500 μL of 0.25% EDTA-free trypsin for 1 min, centrifuged at 1000 g for 5 min, and the supernatant was discarded. The count was resuspended in PBS, and 1× $10^{5}$ cells were taken. Add 195 μL of Annexin V-FITC conjugate, 5 μL of Annexin V-FITC and 10 μL of PI staining solution sequentially, mix well and incubate for 20 min. The cells were detected by flow cytometry.

### 2.10 Cell cycle detection

EMT6 cells were spread in 6-well plates according to the number of 2.5× $10^{6}$ /well, and treated with NBT after growth to 70%–80% for 24 h. The cells were digested with 500 μL of 0.25% EDTA-free trypsin for 1 min, centrifuged at 1000 g for 5 min, and the supernatant was discarded. Add 1 mL of pre-cooled 75% ethanol to resuspend the cells, add 0.5 mL of PI staining solution after 24 h, incubate at 37 °C for 30 min, and then detect the cells by flow cytometry.

### 2.11 Quantitative real-time PCR(qPCR)

Total RNA of each group was extracted with TRIzol reagent (Invitrogen, United States). cDNA was then synthesized by reverse transcription kit (Qiagen, Germany). All experiments were performed according to the instructions provided with the SYBR Premix Ex TaqTM II kit (Takara, China). Quantitative analysis was performed using GAPDH internal standard (Table 1).

**TABLE 1:** Gene primer sequence.

Gene	Primer sequences (5′-3′)
Caspase-1	F: AAT​ACA​ACC​ACT​CGT​ACA​CGT​C
	R: AGC​TCC​AAC​GCT​CGG​AGA​AA
Caspase-3	F:TGACTGGAAAGCCGAAAC
	R:CTGGATGAACCACGACCC
Caspase-8	F:CGTCTATGGAACGGATGGG
	R:TGGCAAGCCTGAATGAAAA
BAX	F:CGTCCACCAAGAAGCTGAG
	R:CAAAGTAGAAGAGGGCAACCAC
BID	F:GCCGAGCACATCACAGACC
	R:TGGCAATGTTGTGGATGATTTCT
Bcl-2	F:TGTGGCCTTCTTTGAGTTCG
	R:TCCCAGCCTCCGTTATCC
P53	F:TTTTCAGGCTTATGGAAACTAC
	R:ACTCGGAGGGCTTCACTT
P21	F:CCTGGTGATGTCCGACCTG
	R:CCATGAGCGCATCGCAATC
CyclinD	F:CGCCCTCCGTATCTTACTTC
	R:TCGCACTTCTGCTCCTCAC
CyclinE	F:TGTGCGAAGTCTATAAGCTCCA
	R:GGCTGAAATCCCAATAAGCTG
GSDMD	F: TTC​AGG​CCC​TAC​TGC​CTT​CT
	R: GTT​GAC​ACA​TGA​ATA​ACG​GGG​TT
GSDME	F: TCA​CCA​CGG​ACA​CCA​ATG​TAG
	R: CCA​CCA​TGT​CTC​CCT​TGG​AG
TNF-α	F:CTGTAGCCCACGTCGTAGCAA
	R:TGTCTTTGAGATCCATGCCGTT
RIPK-1	F:GAAGACAGACCTAGACAGCGG
	R:CCAGTAGCTTCACCACTCGAC
RIPK-3	F:ACCGAATCCAATGACAGGG
	R:AGGCAGTAGTTCTTGGTGGT
MLKL	F:AATTGTACTCTGGGAAATTGCCA
	R:AAAGACTCCTACCGTCCACAG
NLRP3	F: ATT​ACG​CGC​CCG​AGA​AAG​G
	R: CAT​GAG​TGT​GGC​TAG​ATC​CAA​G
MMP-9	F: GGG​ACC​ATC​ATA​ACA​TCA​CAT​AC
	R: TGC​TCC​GCG​ACA​CCA​AAC​T
CCL-5	F: GGA​CAC​CAC​TCC​CTG​CTG​CTT
	R:ACACTTGGCGGTTCCTTCGA
Tgfb3	F: CTC​TCT​GTC​CAC​TTG​CAC​CA
	R: TCA​AGA​TCT​GTC​CCC​TAA​TGG​C
Zbp1	F:GGCAGAAGCTCCTGTTGACT
	R:TATGCTCCATGTTGCAGGCT
GAPDH	F:AGAGTGTTTCCTCGTCCCG
	R:CCGTTGAATTTGCCGTGA

### 2.12 Western blotting

Total proteins were extracted by lysis with RIPA lysis buffer and then quantified by BCA kit (Beyotime, China). Equal doses of isolated proteins were electroblotted onto PVDF membranes (Millipore, United States). The membranes were then incubated with 5% skim milk powder. The membranes were incubated with anti Caspase-1 (Biodragon, RM8397, 1:2000), Caspase-3 (Biodragon, RM8525, 1:2000), Caspase-8 (Biodragon, RM8526, 1:5000), BAX (Biodragon, RM8144, 1:2000), BID (Biodragon, BD-PT0488, 1:1000), Bcl-2 (Biodragon, RM8050, 1:2000), Cyt-c (Biodragon, RM8125, 1:2000), RIPK1 (ABclonal, A7414, 1:1000), RIPK3 (ABclonal, A5431SP, 1:5000), p-RIPK3 (Proteintech, 87148-1-RR, 1:2000), MLKL (ABclonal, A5579, 1:2000), p-MLKL (ABclonal, AP0949, 1:1000), GSDMD (Proteintech, 20770-1-AP, 1:5000), GSDME (Proteintech, 13075-1-AP, 1:2000) and ZBP1 (ABclonal, A13899SP, 1:5000), antigens were treated at 4 °C overnight. After rinsing three times with Tris-Buffered Saline and 0.1% Tween 20, HRP-conjugated Goat anti-Mouse IgG (H + L) (ABclonal, AS003, 1:10,000) or HRP-conjugated Goat anti-Rabbit IgG (H + L) (ABclonal, AS014, 1:10,000) was added and further incubated for 2 h. The proteins were then detected by chemiluminescent ECL reagent (Beyotime) and quantified by β-actin internal standard using ImageJ software.

### 2.13 Bioinformatic analysis

#### 2.13.1 Transcriptome sequencing analysis

The cells were treated with NBT when they grew to 80%–90%. After 24 h of dosing treatment, PBS was washed 2 times. Add 1.2 mL of Trizol and transfer the cell suspension into a 2.5 mL freezing tube. The labelled cryostat tubes were stored directly in liquid nitrogen. Dry ice transportation was used to deliver to the company for sequencing. The sequencing test was based on the Illumina Novaseq 6000 sequencing platform. Library construction was performed using the “SMART-Seq_V4 Ultra-Low-Input RNA Sequencing Kit.” Transcriptome sequencing data were analyzed using the bioinformatics analysis cloud platform provided by Meiji Sequencing (https://cloud.majorbio.com). Gene data mining was performed following raw data sequencing and quality control, reference genome alignment, and transcript assembly. After obtaining read count data from expression analysis, DESeq2 software was used to perform statistical analysis of differentially expressed genes between the Treated and Control groups. The screening criteria were pAdjust <0.05 and |log2FC| ≥ 1, and the BH method was used for multiple comparison correction of p-values.

#### 2.13.2 Protein–protein interaction (PPI) network

Differential genes were analyzed using the STRING online database (https://cn.string-db.org), and the species was selected as Mus_musculus with a confidence level of 0.4 to construct the PPI graph. Key nodes were calculated by Degree centrality, and the top 15 key nodes were selected. They were analyzed for sub-network protein clustering using MCODE plug-in in Cytoscape software.

### 2.14 Establishment of an EMT6 mouse breast cancer xeno-transplant model and drug-grouped treatment

Thirty-two female BALB/C mice, 6–8 weeks, 18–22 g, were purchased from Liaoning Changsheng Biological Company. They were housed in SPF-grade animal rooms. The housing environment complies with the requirements of the Regulations on the Management of Laboratory Animals. Follow-up experimental studies will be conducted after 7 days. Mice were divided into CON group (n = 5), MOD group (n = 5), NBT group (5 mg/kg,n = 5), and PTX group (10 mg/kg,n = 5). Based on the experimental method of Pero et al. (41,942,529). and in combination with the pre-experiment data from our previous studies, we finally completed the modeling plan. Cells were digested, centrifuged and counted, diluted to 1.2 × $10^{6}$ cells/mL. Mice in the CON group were inoculated with 0.1 mL of PBS buffer at the left fourth breast pad, and mice in the other groups were inoculated with tumor cells at the fourth breast pad on the left side of the mice at 0.1 mL/pc. Transplanted tumors were visible to the naked eye at the inoculation site after 7 days. Preliminary judgment was made that the modelling was successful. NBT is sourced from the Ministry of Education Key Laboratory of Zoonotic Diseases at Shenyang Agricultural University. As determined by HPLC, its certified purity is ≥ 98.5%. The stock solution was prepared by thoroughly dissolving 1 mg of Neobractatin in 1 mL of DMSO, then diluting to a final volume of 2.1552 mL with RPMI-1640 medium containing 5% fetal bovine serum. The mixture was thoroughly shaken to ensure homogeneity, yielding a Neobractatin stock solution with a final concentration of 1 mM. The stock solution was aliquoted and stored at 4 °C. PTX was purchased from Sigma with an HPLC-determined purity of ≥97%. It was dissolved in DMSO to prepare a 10 mg/mL stock solution and stored at −20 °C. The drug was administered via intraperitoneal injection once daily for 14 days, with a single injection volume of 0.1 mL. Based on body weight measured on the day of each injection, mice were administered 5 mg/kg NBT or 10 mg/kg PTX, respectively. The drug solvent consisted of 2% DMSO +40% PEG 300 + 2% Tween 80 + ddH_2_O. Mice in the CON group received an intraperitoneal injection of 0.1 mL saline, while mice in the MOD group received an intraperitoneal injection of 0.1 mL drug solvent. The volume of the transplanted tumor, V, was calculated as V = 0.52 × a × b^2^, where V represents the volume of the transplanted tumor, a represents the major axis of the tumor, and b represents the minor axis of the tumor.

### 2.15 HE staining

The Tumor tissue were carefully removed and post-fixed in 4% paraformaldehyde for 12 h. Fixed tissues were dehydrated with an automatic dehydrator (Leica HistoCore PEGASUS, Wetzlar, Germany), embedded and sectioned. Hematoxylin–eosin (H&E) staining was performed by using an H&E staining kit (Solarbio, Beijing, China) in accordance with the manufacturer’s instructions. Acquire images using an inverted microscope. Images (400X) were collected of the area of interest to observe pathological changes.

### 2.16 Serum ALT, AST, Cr and BUN detection

Blood was collected by removing the eyeballs, and the blood collection tube was tilted at room temperature for 1 h, then centrifuged at 1000r for 15 min. The supernatant was taken and used for the test immediately, and the rest of the supernatant was put in the refrigerator at −80 °C for storage. Refer to the instructions of Nanjing jiancheng Bioengineering Institute for ALT, AST, Cr and BUN test kit to complete the establishment of the standard curve and the detection of content.

### 2.17 Statistical analysis

All the experimental data in this experiment were significantly analyzed by IBM SPASS Statistics 25 statistical analysis software, and the experimental result graphs were plotted by Origin 2018, Graphpad Prism 8.0.1, and ModFit LT5.0 software. All experiments were repeated with three independent replicates. All the data were normally distributed, and the results are expressed as the means ± standard deviations (SDs). Since all the results follow a normal distribution, therefore based on the parameter assumptions. The statistical significance of the differences between two groups was measured by an unpaired Student’s t-test, and among three or more groups by one-way ANOVA. The difference with the P-value of less than 0.05 was considered statistically significant.

## 3 Results

### 3.1 NBT inhibited the proliferation of EMT6 cells

**FIGURE 1:**
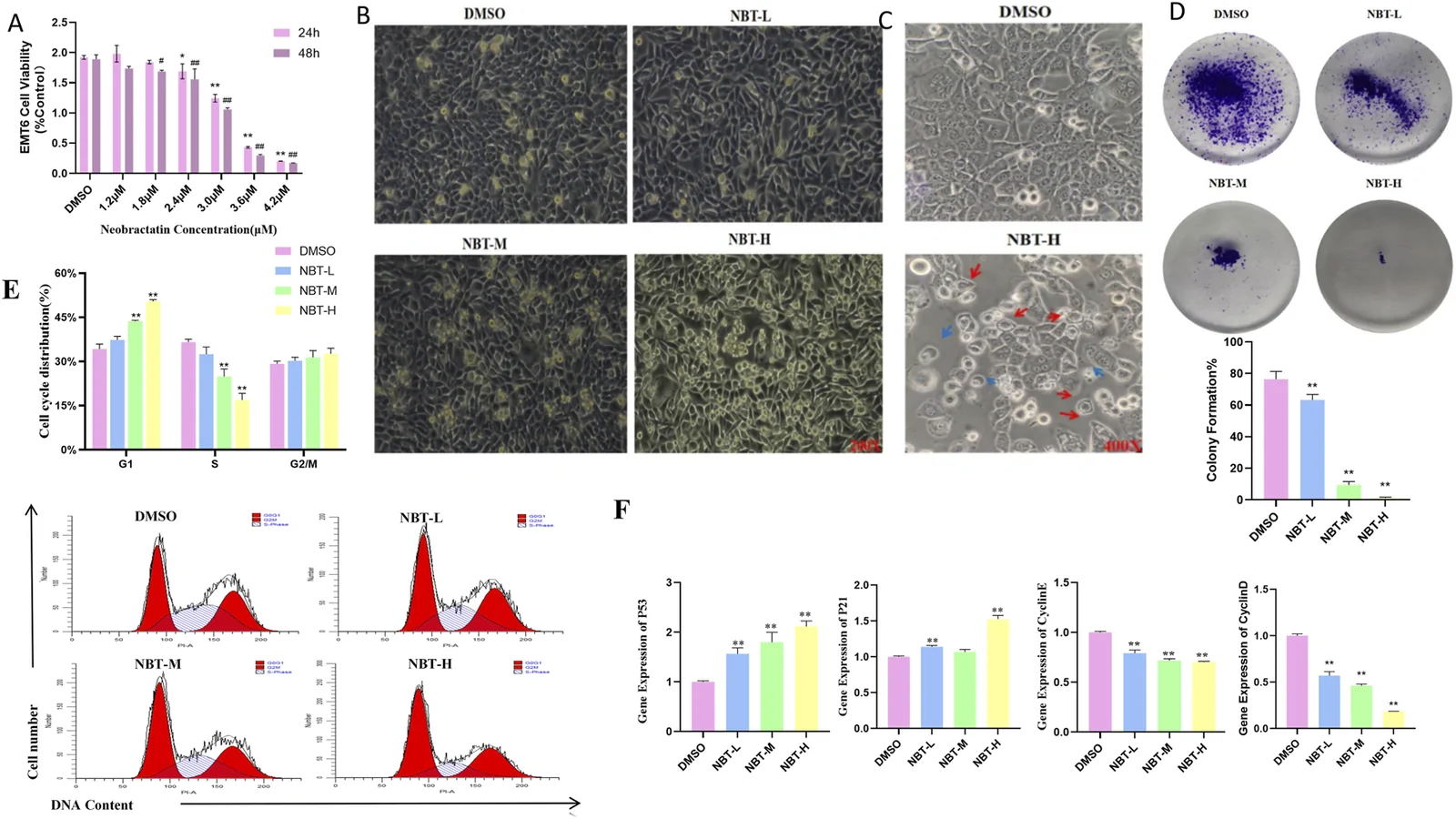
NBT inhibited the proliferation of EMT6 cells. **(A)** Effects of NBT on EMT6 cell viability. **(B)** Effects of NBT on EMT6 cell morphology (200x). **(C)** Effects of NBT on EMT6 Cell morphology (400x). **(D)** Effects of NBT on EMT6 Cell colony formation. **(E)** The flow chart of the cell cycle. **(F)** Effect of NBT on the relative expression of EMT6 cell cycle pathway-related factor mRNA.*p < 0.05, **p < 0.01, ^#^p < 0.05, ^##^p < 0.01 was considered statistically significant with DMSO group.

The CCK-8 assay was used to detect the inhibitory effect of NBT on EMT6 cell proliferation. The NBT group significantly reduced EMT6 cell viability (Figure 1A). Electron microscopy revealed that cells in the DMSO group exhibited irregular triangular shapes with irregular edges and corners, and showed tight connections between adjacent cells. Some cells shrivelled and rounded, with some detaching and suspending (200x) (Figure 1B). At 400x magnification, EMT6 cells in the NBT-H group appeared rounded, with most cytoplasms exhibiting vacuolated morphology and some cells displaying bubbles on the membrane surface (Figure 1C). Clonogenic assay revealed that the proliferation capacity of EMT6 cells progressively decreased with increasing NBT concentrations (Figure 1D). Cell cycle analysis showed that, the NBT groups exhibited increased proportion of cells in the G1 phase and reduced proportion in the S phase (Figure 1E). Relative mRNA expression levels of cell cycle-related factors were detected by qPCR. NBT significantly increased the relative expression of the p53 and p21 while decreasing the relative expression of the Cyclin D and Cyclin E (Figure 1F). This indicates that NBT induced G1 phase arrest in EMT6 cells.

### 3.2 NBT induces apoptosis in EMT6 cells

**FIGURE 2:**
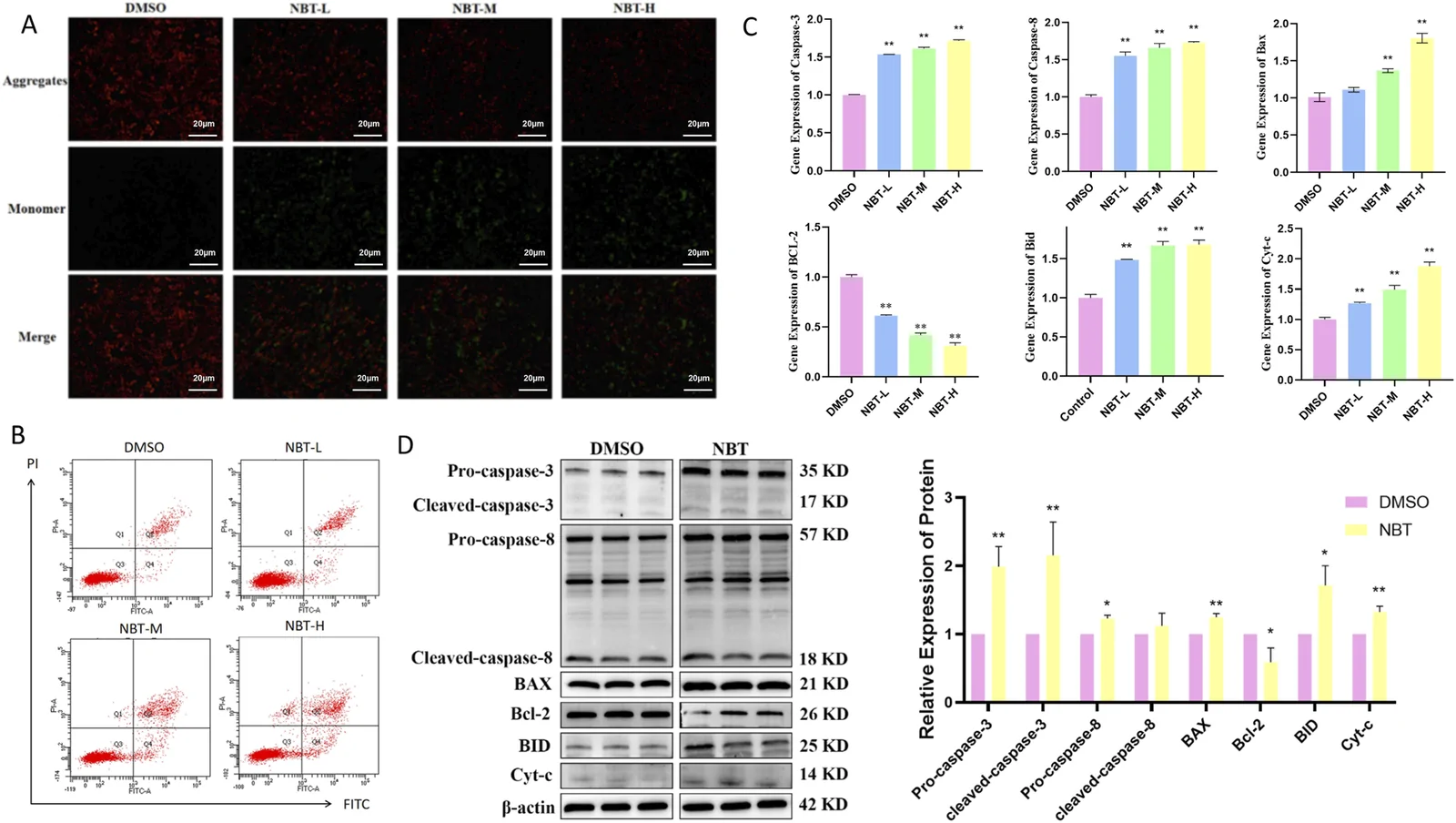
NBT induces apoptosis in EMT6 cells **(A)** Effects of NBT on EMT6 Cell MMP. **(B)** The flow cytometry of apoptosis of EMT6 cells treated with NBT. **(C)** Effect of NBT on the relative expression of mRNA related to the apoptosis pathway in EMT6 cells. **(D)** Effect of NBT on the expression of key proteins in the EMT6 cell apoptosis pathway. *p < 0.05, **p < 0.01 was considered statistically significant with DMSO group.

JC-1 staining revealed that the NBT group exhibited increased green fluorescence and a significant decrease in mitochondrial membrane potential (Figure 2A). Flow cytometry analysis showed that compared the ratios of early apoptotic cells and necrotic cells increased in all NBT concentration groups (Figure 2B). Furthermore, qPCR analysis of key apoptosis-related markers revealed that NBT elevated expression of caspase-3, caspase-8, BAX, Bcl-2, and Cyt-c, while decreasing BID expression (Figure 2C). Western blotting results showed that NBT increased the expression of caspase-3, cleaved caspase-3, caspase-8, cleaved caspase-8, BAX, Bcl-2, and Cyt-c, while decreasing the expression of BID (Figure 2D).

### 3.3 NBT induces pyroptosis in EMT6 cells

**FIGURE 3:**
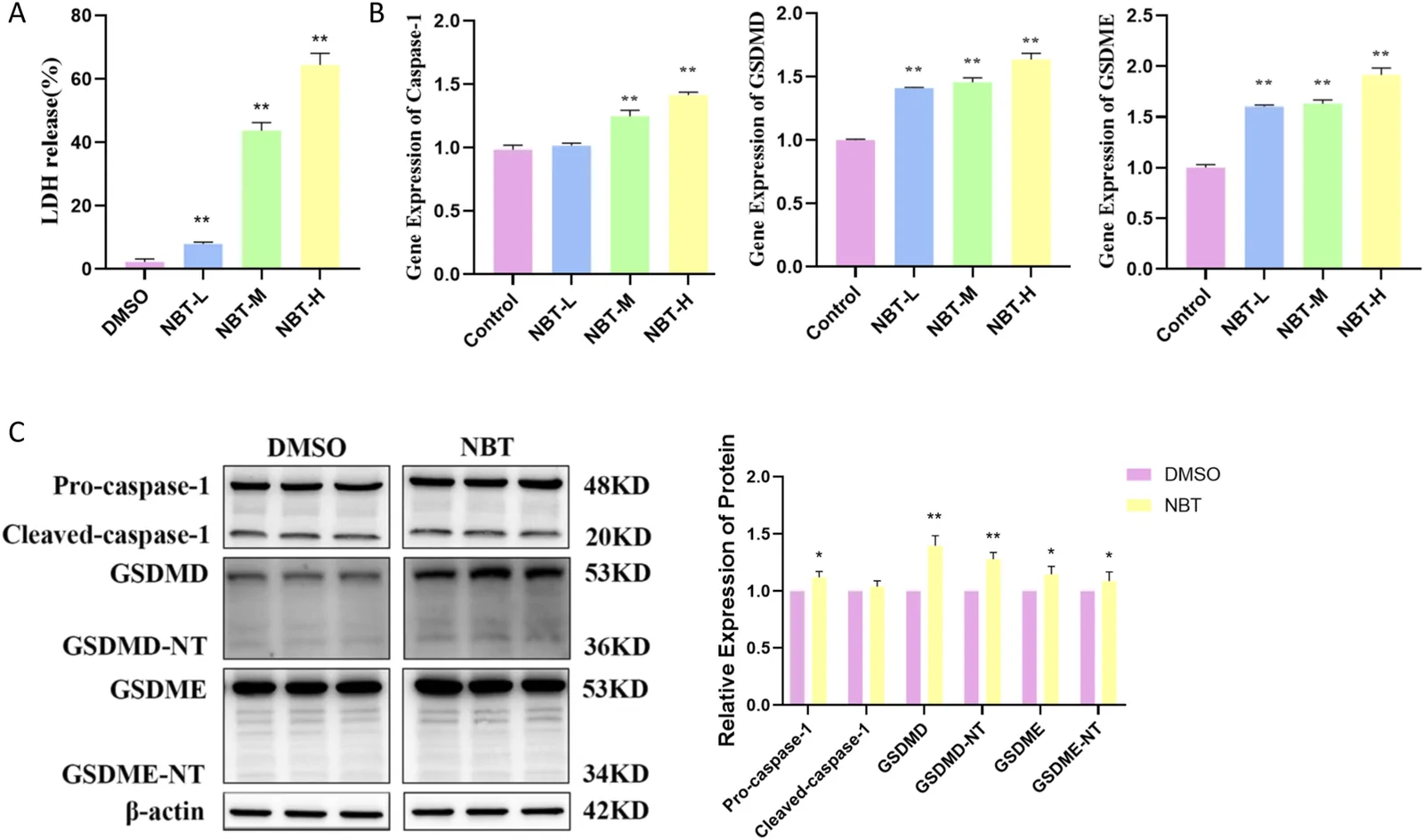
NBT induces pyroptosis in EMT6 cells. **(A)** Effect of NBT on LDH release rate of EMT6 cells. **(B)** Effect of NBT on the relative expression of mRNA related to the pyroptosis pathway in EMT6 cells. **(C)** Effect of NBT on the expression of key proteins in the EMT6 cell pyroptosis pathway. p > 0.05 (ns) represented insignificant differences; *p < 0.05, **p < 0.01 was considered statistically significant with DMSO group.

NBT increased LDH release in EMT6 cells in a concentration-dependent manner (Figure 3A). Key pyroptosis-related markers in EMT6 cells were detected by qPCR. Compared with the DMSO group, mRNA expression of Caspase-1, GSDMD, and GSDME was significantly elevated in the NBT group (Figure 3B). Western blotting results demonstrated that NBT increased the expression of Caspase-1, cleaved caspase-1, GSDMD, GSDMD-NT, GSDME, and GSDME-NT (Figure 3C).

### 3.4 NBT induces necroptosis in EMT6 cells

**FIGURE 4:**
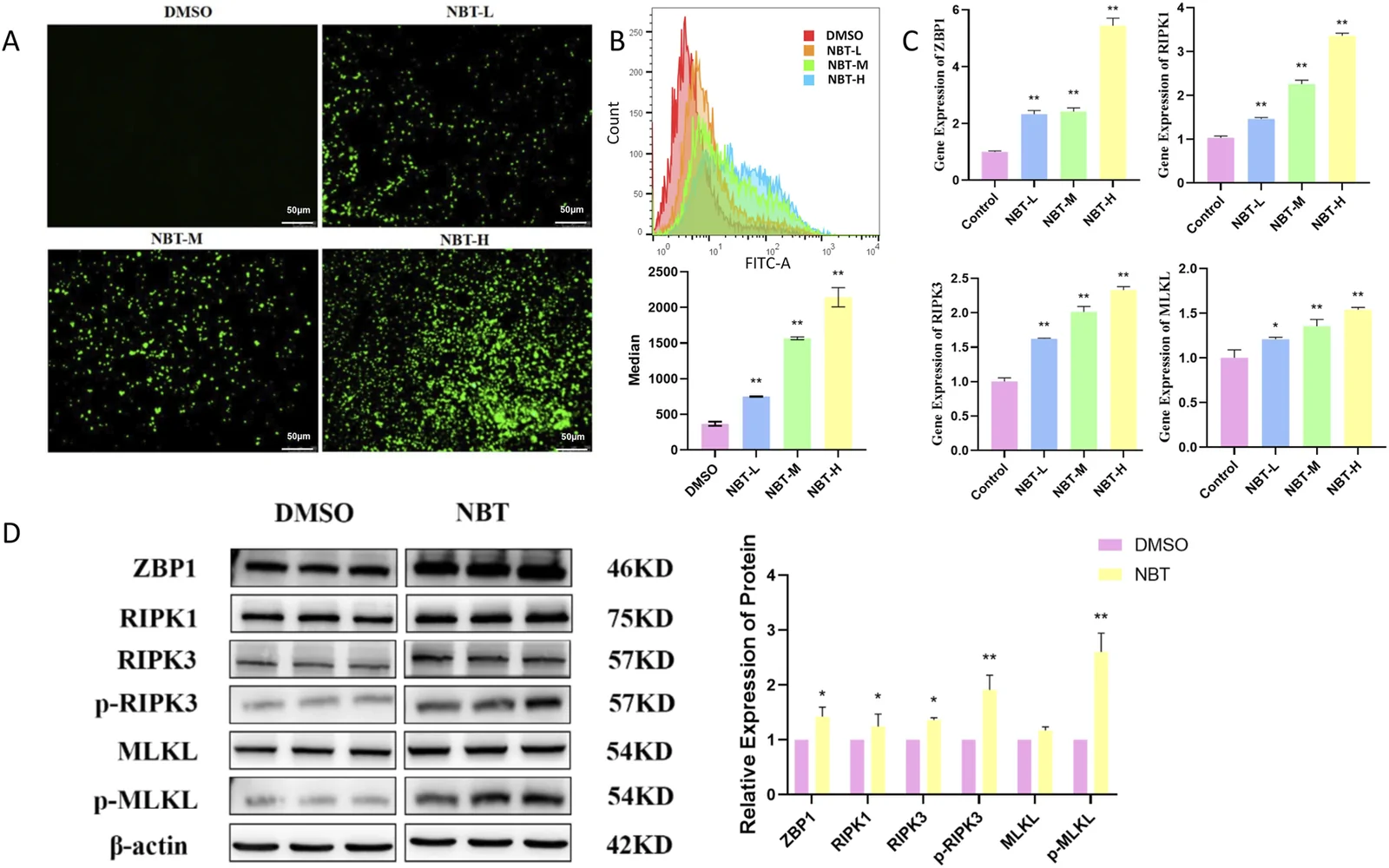
NBT induces necroptosis in EMT6 cells. **(A)** Effects of NBT on EMT6 Cell ROS. **(B)** Flow cytometry shows changes in ROS. **(C)** Effect of NBT on the relative expression of mRNA related to the necroptosis pathway in EMT6 cells. **(D)** Effect of NBT on the expression of key proteins in the EMT6 cell necroptosis pathway. *p* > 0.05 (ns) represented insignificant differences; *p < 0.05, **p < 0.01 was considered statistically significant with DMSO group.

DCFH-DA staining and flow cytometry analysis of ROS revealed increased ROS production with escalating NBT concentrations (Figures 4A,B). q-PCR detected expression of key markers associated with necroptosis in EMT6 cells. Compared to the DMSO group, mRNA expression of ZBP1, RIPK1, RIPK3, and MLKL was significantly elevated in the NBT group (Figure 4C). Western blotting analysis demonstrated that NBT treatment increased the expression of ZBP1, RIPK1, RIPK3, p-RIPK3, MLKL, and p-MLKL (Figure 4D).

### 3.5 Analysis of transcriptome sequencing results

**FIGURE 5:**
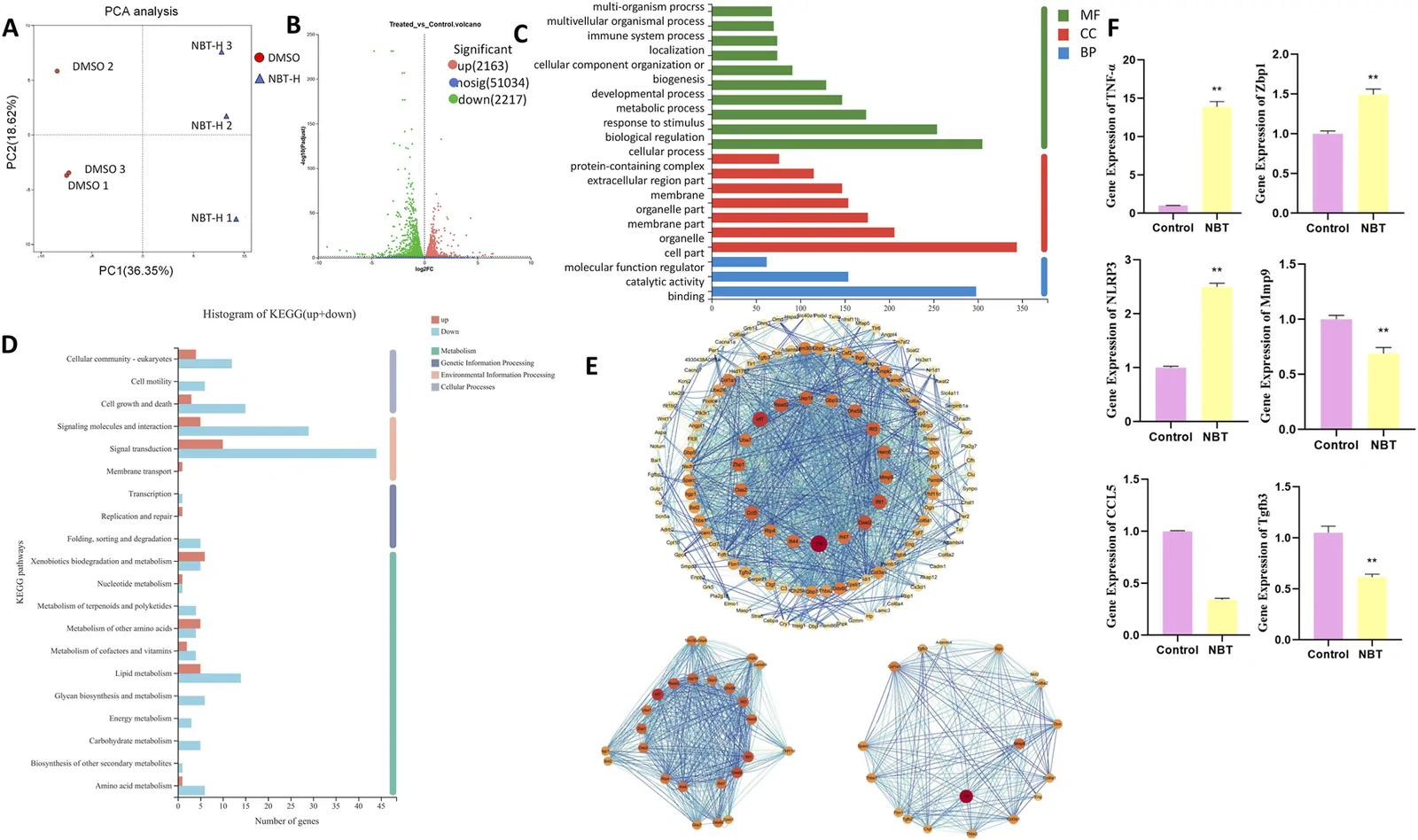
Analysis of transcriptome sequencing results. **(A)** PCA Venn analysis. **(B)** The volcano diagram of Treated vs. Control expression difference. **(C)** The result diagram of GO annotations analysis. **(D)** The results diagram of KEGG function up. **(E)** The interaction analysis of PPI in NBT vs. DMSO group; Figure B and C show the protein interaction analysis of sub networks. **(F)** qPCR verification of differentially expressed genes. **p < 0.01 was considered statistically significant with DMSO group.

PCA analysis showed that there was good biological duplication between the DMSO group and the NBT group (Figure 5A). A total of 504 DEGs were detected in the NBT group compared with the DMSO group, of which 115 were upregulated,and 389 were downregulated (Figure 5B). GO functional annotation found that differential genes are mainly concentrated in Cellular process, Biological regulation and Response to stimulus. In terms of Cell components, it is primarily concentrated in the Cell part, Organelle, and Membrane part. In terms of molecular functions, the major abdominal muscles were involved in Binding and Catalytic activity (Figure 5C). The KEGG feature notes found that differential gene sets are mainly annotated to Metabolisms, Genetic Information Processing, Environmental Information Processing and Cellular Processes (Figure 5D). PPI analysis identified 18 differentially expressed genes, including TNF and ZBP1, as key targets (Figure 5E). Randomly selected TNF-α, ZBP1, NLRP3, MMP9, CCL5, and Tgfb3 were validated by qPCR, results consistent with transcriptomic sequencing data (Figure 5F). To further explore the potential roles of regulated cell death, we extracted KO and KEGG pathways associated with apoptosis, pyroptosis, and necroptosis from the enrichment results and visualized them using separate bubble plots (Supplementary Figure S1A,B).

### 3.6 NBT promotes PANoptosis in EMT6 cells via ZBP1

**FIGURE 6:**
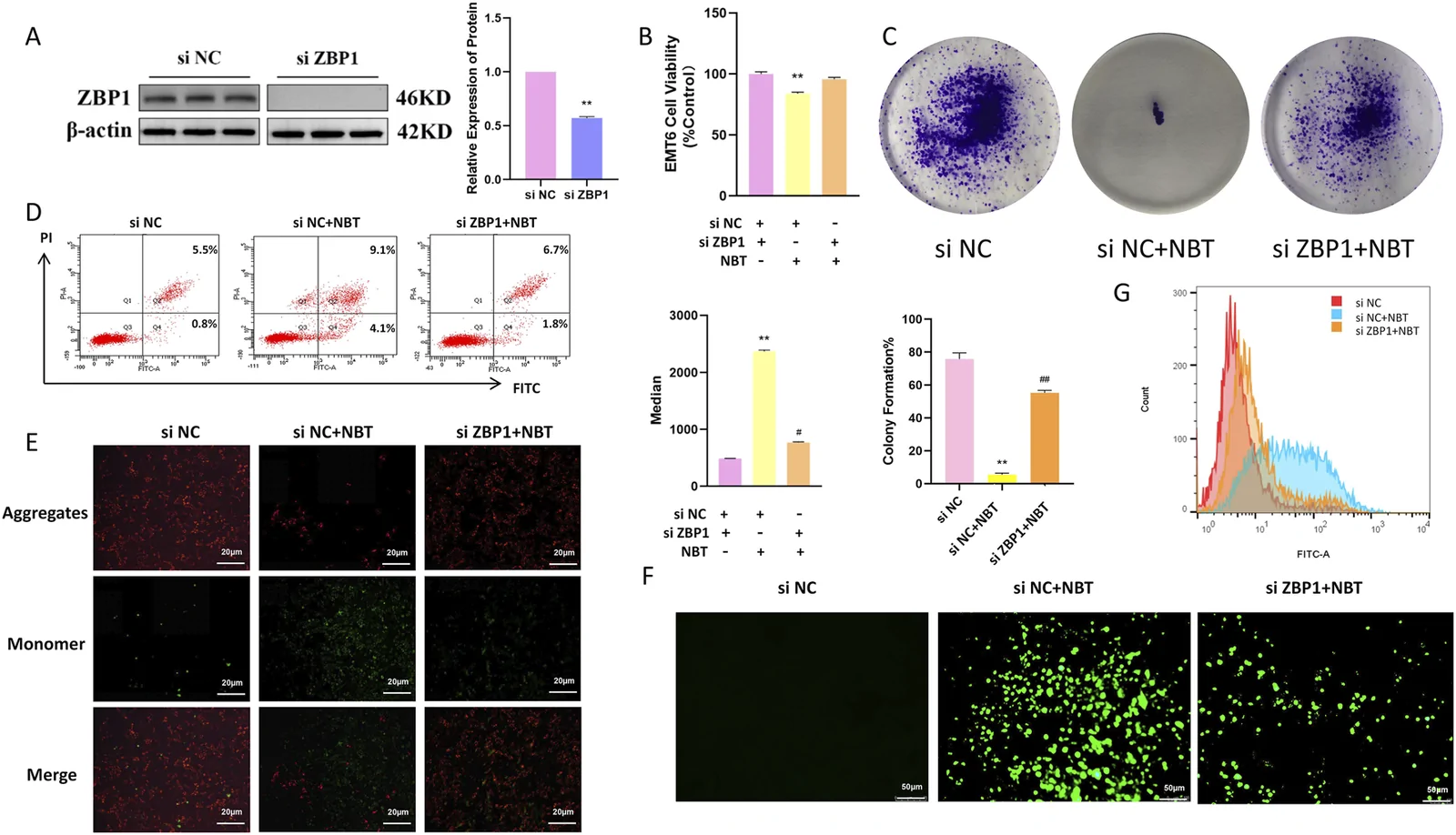
NBT Promotes PANoptosis in EMT6 Cells via ZBP1. **(A)** Western blot analysis was used to assess the efficiency of ZBP1 transfection efficiency. **(B)** Effects of si ZBP1 on EMT6 cell viability. **(C)** Effects of si ZBP1 on EMT6 Cell colony formation. **(D)** The flow cytometry of apoptosis of EMT6 cells treated with si ZBP1. **(E)** Effects of si ZBP1 on EMT6 Cell MMP. **(F,G)** Effects of si ZBP1 on EMT6 Cell ROS. **p < 0.01, ^#^ *p* < 0.05, ^##^ *p* < 0.01 was considered statistically significant with si NC group.

To determine whether the PTE inhibitory effect on breast cancer is mediated by ZBP1, we silenced ZBP1 expression using siRNA. Western blotting analysis confirmed successful reduction of ZBP1 expression (Figure 6A). Compared to the si NC group, NBT significantly reduced cell viability; however, knocking down ZBP1 reversed this inhibitory effect (Figure 6B). Similar trends were observed in the clonogenic assay and flow cytometry (Figures 6C,D). Furthermore, fluorescence microscopy revealed that NBT significantly reduced mmp, and ZBP1 knockdown reversed this decrease (Figure 6E). Fluorescence microscopy and flow cytometry analysis showed that ZBP1 knockdown also reversed NBT-induced increases in ROS levels in EMT6 breast cancer cells (Figures 6F,G). These findings indicate that NBT promotes EMT6 cell PANoptosis by upregulating ZBP1.

### 3.7 NBT inhibits the growth of xenografted breast tumors in mice

**FIGURE 7:**
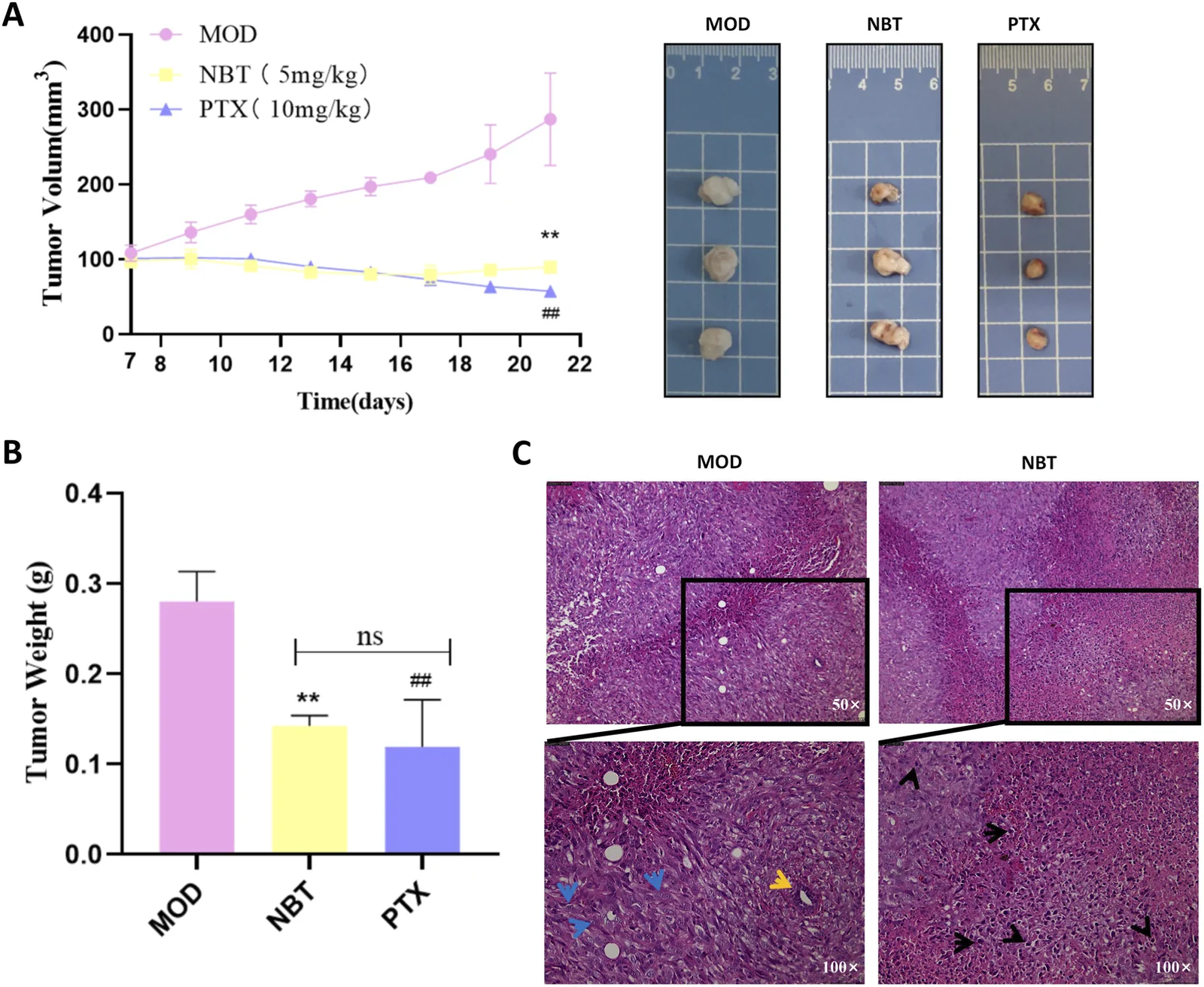
NBT Inhibits the growth of Xenografted Breast Tumors in mice. **(A)** Tumor growth curve and final tumor volume in each group. **(B)** Appearance of transplanted tumors. **(C)** Histopathological results of transplanted tumors. **p < 0.01,^##^ *p* < 0.01 was considered statistically significant with MOD group.

Based on xenograft volumes in each experimental group, at day 21 post-tumor implantation, both the NBT and PTX groups exhibited significantly reduced tumor volumes compared to the MOD group (Figure 7A). Compared to the MOD group, both the NBT and PTX groups showed decreased xenograft weights (Figure 7B). HE staining revealed that the MOD group exhibited the highest number of tumor cells, which were densely packed and frequently showed mitotic figures. In contrast, the NBT group demonstrated fewer tumor cells compared to the MOD group, with nuclei beginning to dissolve and necrotic cells appearing (Figure 7C). This means NBT Inhibits the growth of Xenografted Breast Tumors in mice.

### 3.8 Effect of NBT on PANoptosis of transplanted breast cancer *in vivo*

**FIGURE 8:**
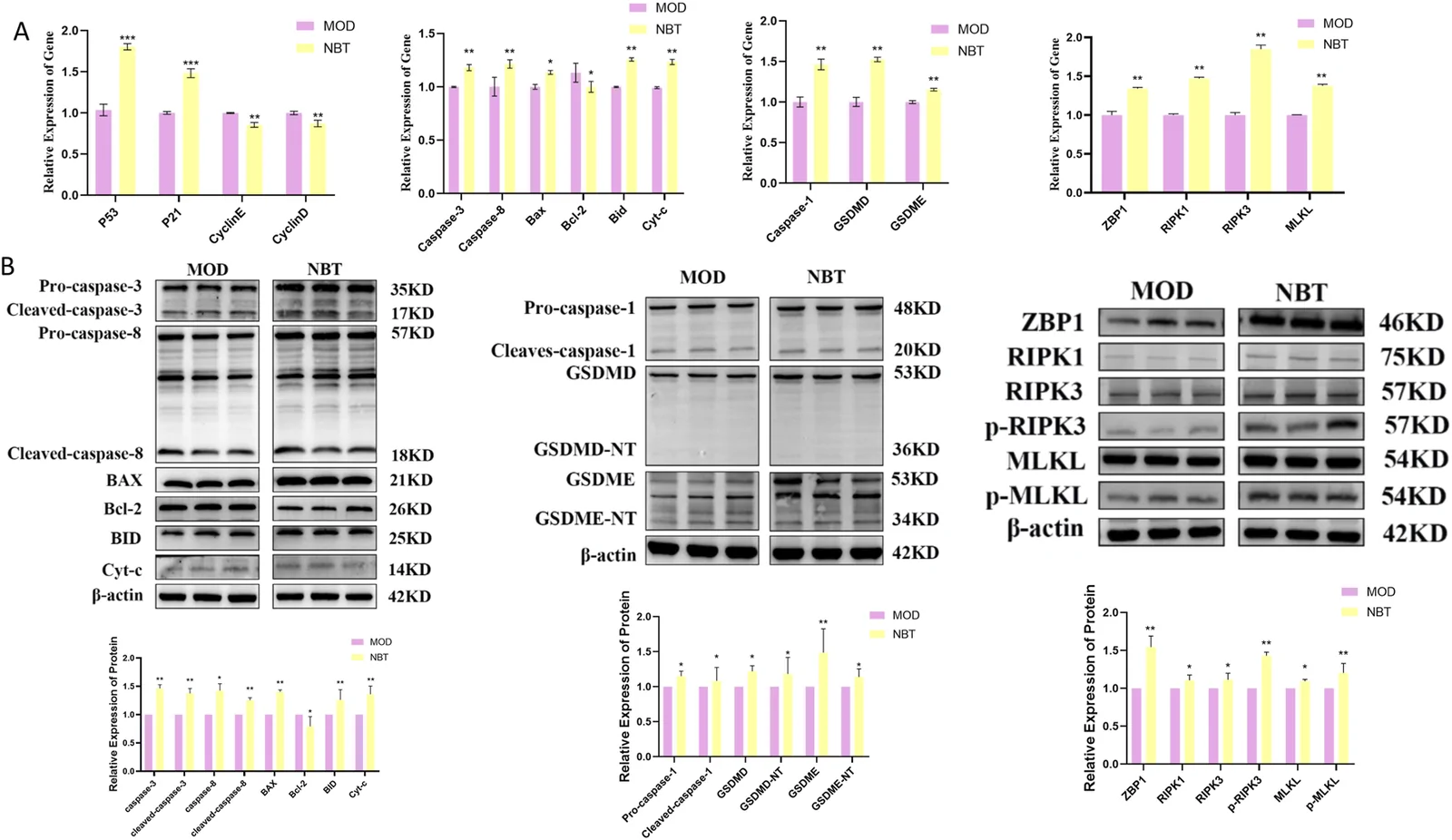
Effect of NBT on PANoptosis of transplanted breast cancer *in vivo*. **(A)** Effect of NBT on the relative expression of mRNA related to PANoptosis pathway in EMT6 cells. **(B)** Effect of NBT on the expression of key proteins in the EMT6 cell PANoptosis pathway. *p < 0.05, **p < 0.01, ***p < 0.001 was considered statistically significant with MOD group.

PCR detected expression of key markers associated with PANoptosis in EMT6 cells. Compared to the MOD group, mRNA expression of P53, P21,caspase-3, caspase-8, BAX, Bcl-2, Cyt-c, Caspase-1, GSDMD, GSDME, ZBP1, RIPK1, RIPK3, and MLKL, while decreasing BID, cyclin E and cyclin D expression (Figure 8A). Western blotting results showed that NBT increased the expression of caspase-3, cleaved caspase-3, caspase-8, cleaved caspase-8, BAX, Bcl-2, Cyt-c, Caspase-1, cleaved caspase-1, GSDMD, GSDMD-NT, GSDME, GSDME-NT, ZBP1, RIPK1, RIPK3, p-RIPK3, MLKL, and p-MLKL while decreasing the expression of BID (Figure 8B). This indicates that NBT exerts its anti-breast cancer effects *in vivo* through the PANoptosis pathway.

### 3.9 Evaluation of *in vivo* safety of NBT

**FIGURE 9:**
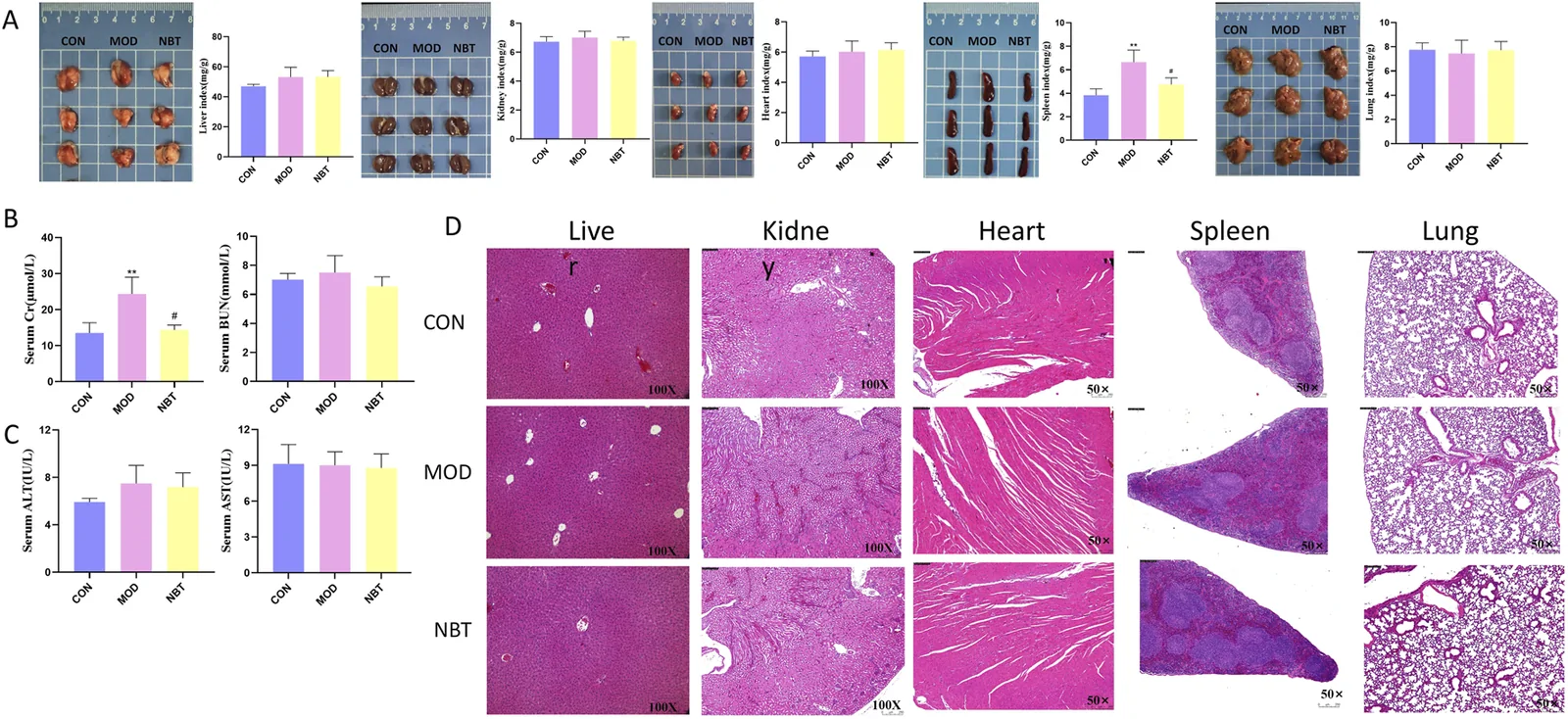
Evaluation of *in vivo* safety of NBT. **(A)** The liver, kidney, heart, spleen and lung pictures of mice. **(B)** The serum Cr and BUN content of mice. **(C)** The serum ALT and AST content of mice. **(D)** HE staining of the mouse liver, kidney, heart, spleen and lung. **p < 0.01, ^#^ *p* < 0.05 was considered statistically significant with MOD group.

No macroscopic pathological changes were observed in the livers, kidneys, hearts, spleens, and lungs of mice in the CON, MOD and NBT groups (Figure 9A). HE staining revealed no pathological lesions in the sections from any group (Figure 9B). Compared to the CON group, serum levels of AST, ALT, and BUN showed no significant changes in the MOD and NBT groups. Compared to the MOD group, serum Cr levels were significantly reduced in the NBT group (Figure 9C). These results indicate that NBT exhibits low toxicity and does not cause organ damage at the therapeutic dose of 5 mg/kg.

## 4 Discussion

NBT is a compound extracted from Garcinia bracteata C. Y. Wu, serving as a food–medicine homology substances with anti-inflammatory and anti-tumor effects (Tan et al., 2025). Studies have shown that NBT can inhibit the growth of esophageal cancer, cervical cancer, liver cancer, human brain glioma cells and breast cancer cells, but the related mechanism of its role in breast cancer is still unclear (Zhang et al., 2019; Li Z. et al., 2024; Xue et al., 2020). In our study, We found that NBT treatment resulted in concentration-dependent, graded phenotypic changes. Low concentrations of NBT only slightly inhibited tumor cell growth and did not induce significant cell death. At moderate concentrations, cells exhibited a marked G1/S phase cell cycle arrest, thereby inhibiting continued cell proliferation. Treatment with high concentrations further triggered intense PANoptosis, resulting in maximum antitumor activity. In addition, we found the morphology of EMT6 cells treated with NBT showed that swelling and vacuum-like morphological change in the cytoplasm before shrinking and falling off. In addition, a small number of cells were swollen and accompanied by the formation of bubbles on the plasma membrane surface. This means that EMT6 cells treated with NBT do not simply conform to the morphological changes of single apoptosis, pyroptosis and necroptosis. Seems to be undergoing a PANoptosis form of death (Chen et al., 2016; Thompson, 1998; Vande Walle et al., 2016). To further validate this hypothesis, we combined RNA-seq and experimental to investigate NBT-induced PANoptosis in breast cancer.

Post-transcriptional regulation is an essential regulation of gene expression and signal transmission in the body, regulating cell proliferation, differentiation, metastasis and death. According to RNA-seq analysis, a total of 504 DEGs were detected in the NBT group compared with the DMSO group. Further functional enrichment analysis of GO and KEGG genes related to shared differential genes and biological regulation and signal transduction showed that differential genes were mainly enriched in TNF signaling pathway and MAPK signaling pathway. Co-treatment with IFN-γ and TNF-α induces pan-apoptosis in dMMR tumor cells and reduces drug resistance (Li H. et al., 2024). Paclitaxel and cephalomannine promote PANoptosis in TNBC cells by regulating ROS through TNF-α (Gao et al., 2025). This aligns with our RNA-seq findings, which show that NBT promotes EMT6 cell PANoptosis by activating TNF-α. MAPK has been found in breast cancer to promote invasion, metastasis and epithelial mesenchymal transformation of breast cancer cells through stromal amplification (Hong et al., 2015; Ke et al., 2021; Wen et al., 2019). PPI analysis showed that TNF and ZBP1 was the most critical protein node in the network, which was also corresponding to the KEGG enrichment results. Research has shown that KLX ameliorates liver cancer progression by mediating ZBP1 transcription and ubiquitination and increasing ZBP1-induced PANoptosis (Wang et al., 2024). Additionally, a study published in December 2025 found that activating ZBP1 enhances the efficacy of immunotherapy in non-small cell carcinoma (Xu et al., 2025). It is known that ZBP1 is a key initiator of PANoptosis (Wang Z. et al., 2025), but the regulatory mechanism of NBT on ZBP1 in breast cancer remains unclear. Our study indicates that si ZBP1 attenuates the cytotoxic effect of NBT on EMT6 cells, suggesting that ZBP1-induced PANoptosis is a key mechanism.

Alternative mechanisms that simultaneously drive multiple programmed cell death pathways can effectively eliminate cancer cells (Markiewicz et al., 2021). When apoptosis is ineffective, activation of pyroptosis and necroptosis is conducive to inhibiting the growth of cancer cells. It has been shown that both inflammatory vesicles and caspase-8/RIPK3 can trigger PANoptosis as components of the panoptosome (Wang et al., 2023; Xiong, 2023). ZBP1 has been shown to initiate PANoptosis by interacting with RIPK3 (Huang et al., 2025). The ZBP1-PANoptosome, first discovered by the Kanneganti laboratory, acts as a molecular scaffold that simultaneously activates pyroptosis, apoptosis, and necroptosis pathways, representing a unified inflammatory cell death program (Bhardwaj et al., 2026). It is known that TNF-mediated signaling induces a dynamic transition of cells from a survival state to a death state: membrane-associated complex I promotes NF-κB activation through RIPK1 ubiquitination, while deubiquitination leads to the formation of cytoplasmic complex II, which contains caspase-8 and can drive either apoptosis or necroptosis when caspase-8 function is impaired (Brenner et al., 2015). However, the relationship between TNF/RIPK1 signaling and ZBP1-mediated pan-apoptosis remains unclear. Recent evidence suggests that RIPK1 is a key regulator of ZBP1-RIPK3 complex activation. Jiang et al. demonstrated that during PANoptosis, RIPK1 promotes the phosphorylation of RIPK3 and facilitates the interaction between ZBP1 and RIPK3 (Lu et al., 2026). Furthermore, studies have shown that the interaction between ADAR1 and ZBP1 modulates the threshold for Z-DNA-induced cell death, with ADAR1 limiting ZBP1 activation during the early stages. Based on these findings, we propose a hierarchical model: NBT-induced TNF upregulation provides an initial initiating signal through the scaffolding role of RIPK1, which in turn enables ZBP1 to recruit RIPK3 and assemble the PANoptosome, ultimately driving GSDMD-mediated PANoptosis. PANoptosis can impede immune escape, and activation of panoptosis in tumour cells can help to prevent them from becoming immunosuppressed (Zhou et al., 2024). Although apoptosis, pyroptosis, and necroptosis have been extensively studied in breast cancer, there is still a significant gap in the study of PANoptosis in breast cancer. Currently, we have conducted preliminary studies on a few key regulatory targets of NBT in promoting PANoptosis in breast cancer, such as upstream molecules ZBP1, RIPK1, RIPK3, caspase family members (including Caspase-3 and Caspase-8), and downstream molecules. Overall, these data suggest that NBT sensitizes breast cancer cells, causing them to undergo apoptosis, pyroptosis and necroptosis, suggesting that PANoptosis is occurring. However, it is likely that there are likely other molecules dominate the inhibitory effects of NBT on breast cancer.

This study still has some limitations. First, we observed that G1/S-phase cell cycle arrest and PANoptosis occurred simultaneously following NBT treatment. However, the causal relationship between cell cycle arrest and PANoptosis remains unclear. We speculate that this may be due to the induction of cellular stress following NBT treatment, which simultaneously activates independent signaling cascades, leading to both cell cycle arrest and PANoptosis. On the other hand, persistent replication stress caused by G1/S checkpoint arrest may act as an upstream signal promoting the activation of PANoptosis. This will be an important focus of our future research. Second, we confirmed that NBT treatment leads to upregulation of ZBP1 expression, but the upstream molecular mechanism remains unclear. Further experiments are needed to determine whether NBT regulates ZBP1 at the transcriptional, post-transcriptional, or post-translational level, which will help reconstruct the complete regulatory cascade. Third, although we detected changes in the mRNA and protein levels of PANoptosis markers in tumor tissue lysates via qRT-PCR and Western blot, these global detection results cannot reveal the spatial distribution of ZBP1, ZBP1, c-GSDMD, and p-RIPK3 within tumor lesions. Further immunohistochemical analysis is needed to characterize their localization, which will provide more direct visual evidence of PANoptosis activation within the tumor. In summary, the findings of this study offer a highly promising therapeutic strategy for the treatment of breast cancer.

## 5 Conclusion

Our study confirms that NBT may serve as an effective traditional Chinese medicine against breast cancer by promoting PANoptosis through ZBP1 activation. We validated the expression of pyroptosis, apoptosis, and necroptosis molecules in breast cancer cells following NBT treatment and analyzed them through transcriptome sequencing. Additionally, we evaluated the safety profile of NBT. Our findings provide a theoretical basis for the clinical application of NBT in breast cancer treatment.

## Data Availability

The datasets presented in this study can be found in online repositories. The names of the repository/repositories and accession number(s) can be found in the article/Supplementary Material.
