# Supplementary material for: Neobractatin mediated ZBP1 induces PANoptosis of xenografted breast tumors
Source: Front Pharmacol. 2026 Sep 9;17:1913482. doi: 10.3389/fphar.2026.1913482 (PMC13597776; doi:10.3389/fphar.2026.1913482)
Supplement: Supplementary file 1 [file Supplementaryfile1.doc]

**Fig.S1** Bubble plot showing significantly enriched GO and KEGG pathways associated with apoptosis, pyroptosis and necroptosis. (A) GO Enrichment Bubble Chart. (B) KEGG Enrichment Bubble Chart. The horizontal axis represents the rich factor, and the size of each bubble indicates the number of enriched genes.
